# Association Between Long-Term Visit-to-Visit Hemoglobin A1c and Cardiovascular Risk in Type 2 Diabetes: The ACCORD Trial

**DOI:** 10.3389/fcvm.2021.777233

**Published:** 2021-11-24

**Authors:** Dan Huang, Yong-Quan Huang, Qun-Ying Zhang, Yan Cui, Tian-Yi Mu, Yin Huang

**Affiliations:** ^1^Emergency Department, Affiliated Hospital of North Sichuan Medical College, Nanchong, China; ^2^Department of Ultrasound, The Fifth Affiliated Hospital, Sun Yat-sen University, Zhuhai, China; ^3^Department of Geriatrics, The Fifth Affiliated Hospital, Sun Yat-sen University, Zhuhai, China; ^4^Department of Cardiology, The Fifth Affiliated Hospital, Sun Yat-sen University, Zhuhai, China

**Keywords:** cardiovascular risk, mortality, ACCORD 07 trial, diabetes - quality of life, HbA1c - hemoglobin A1c

## Abstract

**Background:** To explore the association between visit-to-visit variability of glycated hemoglobin (HbA1c) and cardiovascular outcomes in the patients with type 2 diabetes mellitus (T2DM) of the Action to Control Cardiovascular Risk in Diabetes (ACCORD) study.

**Methods:** We conducted a *post-hoc* analysis on the ACCORD population including 9,544 participants with T2DM. Visit-to-visit variability of HbA1c was defined as the individual SD, coefficient of variation (CV), and variability independent of the mean (VIM) across HbA1c measurements. The clinical measurements included primary outcome [the first occurrence of non-fatal myocardial infarction (MI), non-fatal stroke or cardiovascular death], total mortality, cardiovascular death, non-fatal MI event, non-fatal stroke, total stroke, heart failure, macrovascular events, and major coronary events (CHD).

**Results:** Over a median follow-up of 4.85 years, 594 and 268 participants experienced all-cause mortality and cardiovascular mortality, respectively. After adjusting for baseline HbA1c levels and confounding factors, the adjusted hazard ratio (HR) comparing patients in the highest vs. the lowest quartile CV of HbA1c variability was 1.61 (95% CI 1.29–2.00) for the primary outcome. Similar trends for secondary outcome were also observed. There was no association between HbA1c fluctuation and non-fatal stroke. Noticeably, there was 66% greater risk for the all-cause mortality among patients in the highest vs. the lowest quartile (HR 1.66, 95% CI 1.27–2.17).

**Conclusions:** Greater variability of HbA1c is associated with higher risk for cardiovascular complications and all-cause death in T2DM. Our study stresses the significance of well-controlled glycemic levels for improving cardiovascular outcomes. Further randomized clinical trials are required to confirm these findings.

## Introduction

Statistically speaking, diabetes was estimated by WHO as the 7th leading cause of mortality, which contributed to 1.6 million deaths in 2016. It has arisen the attention of the world not only because of its growing prevalence but also of increased higher risks for macrovascular and microvascular complications (1–3). Even in the patients with prediabetes, the risk of macrovascular and microvascular disease was increased (4–6). Glycated hemoglobin (HbA1c) presents the average plasma glucose concentration in the past 3 months (7). Recent randomized controlled trials have found that long-term glycemic fluctuation was closely associated with macrovascular and microvascular complications in both type 1 and type 2 diabetes mellitus (T2DM) (8–14). In Diabetes Control and Complications Trial (DCCT), HbA1c levels predicted risk for the renal disease and cardiovascular events in type 1 diabetes (15). A Chinese study contains 91,866 participants demonstrated that HbA1c variability contributed to the development of cardiovascular disease (CVD) and all-cause mortality, particularly in the elderly cohort (16). What is more, ADVANCE study replicated the similar results that visit-to-visit HbA1c fluctuation increased the risk of CVD in T2DM (10). Nevertheless, in the Renal Insufficiency and Cardiovascular Events (RIACE) study, HbA1c variability was associated with macrovascular complications, but not microvascular complication, particularly nephropathy (17). Furthermore, KIM et al. suggested that higher HbA1c fluctuation could not predict carotid artery intima-media thickness independently (18). Besides, participants with diabetes being treated with hypoglycemic agents usually fluctuate greatly in the blood glucose. Therefore, it is important to explore whether the HbA1c variability is an independent risk factor in participants with diabetes. As a result, we performed data analysis in ACCORD cohort to evaluate the association between visit-to-visit HbA1c variability and the risk of cardiovascular outcomes in the patients with T2DM.

## Methods

### Study Design and Participants

The ACCORD trial is a randomized, multicenter, double-blind, 2 × 2 factorial trial, consisting of 10,251 patients with T2DM. The study design and results have been published previously (19, 20). Briefly, the trial recruited participants aged 40 to 79 years who had additional cardiovascular risk factors at 77 North American sites (21). Participants were randomly assigned to achieve intensive glycaemia therapy vs. standard glycaemia therapy (plus either antihypertensive or lipid-lowering intervention). Measurement of HbA1c was recorded every 4 months in the participants for up to 7 years (22). Participants who had < three documented HbA1c measurements and missing confounding data were excluded. Finally, we included 9,544 participants from this study.

### Measurement of HbA1c Visit-to-Visit Variability

HbA1c variability was considered as intra-individual SD across visits (17). We calculated intra-personal CV, SD, and VIM of 7-year follow-up HbA1c in this study. In brief, CV for HbA1c was the ratio of SD and mean to correct for larger SD on account of the higher absolute value of HBA1C. VIM was calculated as 100 × SD/mean^β^, and β means the regression coefficient independent of mean. Moreover, all the participants were divided into four quartiles of HbA1c variability for further analyses.

### Outcomes

The primary outcome includes the first occurrence of non-fatal myocardial infarction (MI), non-fatal stroke or cardiovascular death. Secondary outcomes included MI, non-fatal MI, any stroke, non-fatal stroke, death from any cause, death from a cardiovascular cause, congestive heart failure, and revascularization (23).

### Covariates

Baseline variables include sex, age, race, education, smoking and alcoholic status, BMI, waist, HbA1c, systolic blood pressure (SBP), cholesterol, CVD history, insulin usage, antihypertension, or lipid-lowering medications (24).

### Statistical Analysis

The continuous variables were compared by Kruskal–Wallis or ANOVA test and described as mean ± SD. The categorical variables were compared by chi-squared test and described as a percentage. Kaplan–Meier estimates were used to compare the cumulative incident for the cardiovascular outcome and all-cause mortality within subgroups defined by the variability of HbA1c. Cox proportional hazards regression models were used to calculate adjusted hazard ratios (HR) and 95% CI for CVD outcomes. Subjects were divided into quartiles of CV of HbA1c. Model 1 was adjusted for sex, age, race, duration of DM at baseline. Model 2 was further adjusted for level of education, smoking, alcohol abuse, BMI, and waist at baseline. Model 3 included SBP, heart rate, cholesterol, CVD history, insulin usage, antihypertension, or lipid-lowering medications. Model 4 additionally accounted for the baseline HbA1c levels. All the analyses were performed using SPSS statistic (Version 20.0; IBM Corp., New York, USA) and R software (version 3.4.2; R Foundation for Statistical Computing, Vienna, Austria). A two-sided *P* < 0.05 was considered as statistically significance.

## Results

**Figure 1:**
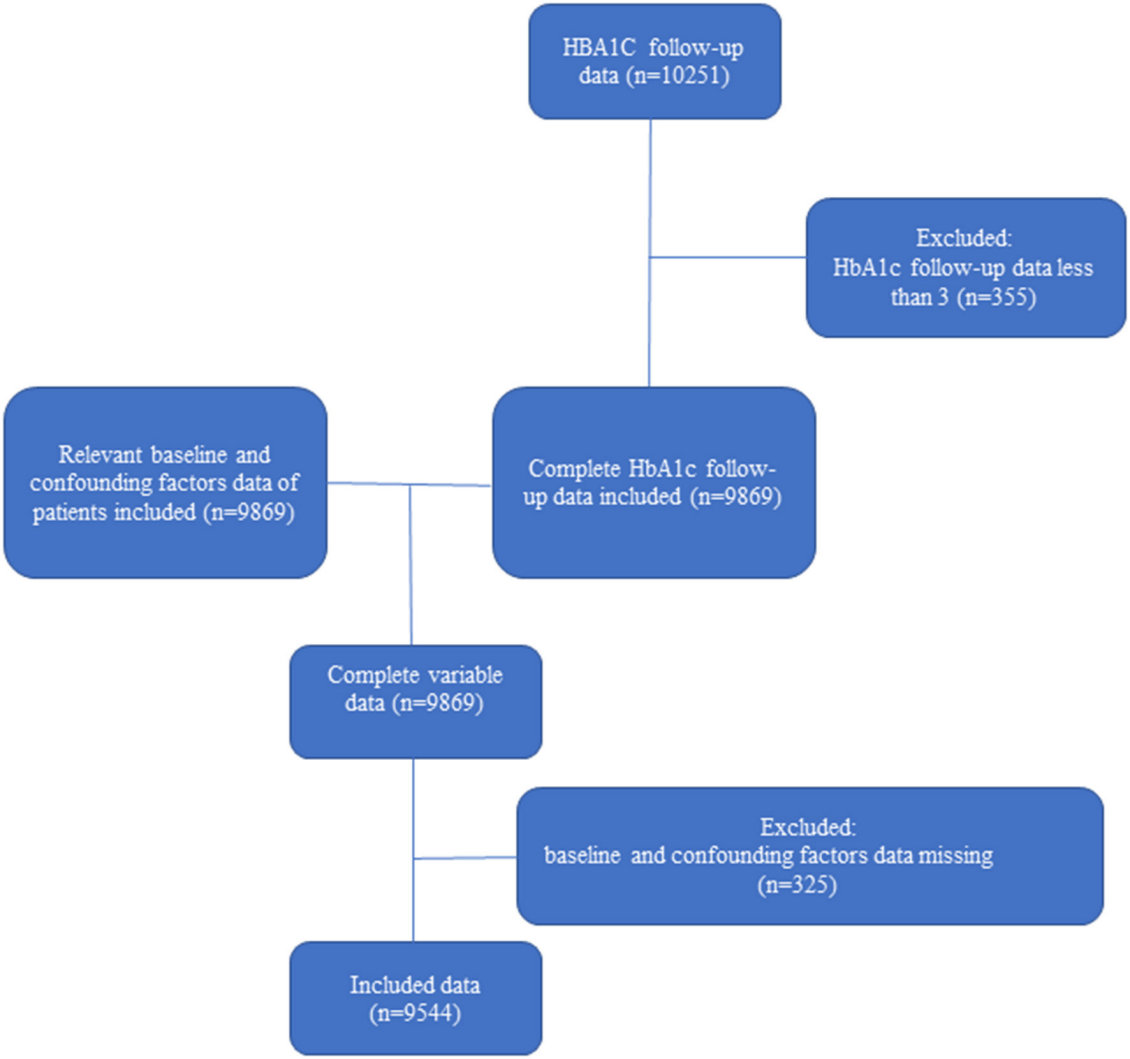
Flow chart of this study.

Figure 1 shows the process of selection of the participants. Of the ACCORD cohort, 9,544 patients included in this study were followed up more than three times and had complete data on the covariates. A proportion of participants with ACCORD were excluded because of missing HbA1c measurements. Baseline characteristics grouped by quartiles of CV are presented in Table 1.

**Table 1:** Baseline characteristics grouped by quartiles of variability of HbA1c.

	**Total**	**Q1**	**Q2**	**Q3**	**Q4**	***p***
		**(0–0.071)**	**(0.071–0.094)**	**(0.094–0.122)**	**(>0.122)**	
Female	3658 (38.3%)	911 (9.5%)	913 (9.6%)	916 (9.6%)	918 (9.6%)	0.997
Age	62.7 ± 6.61	63.88 ± 6.66	63.12 ± 6.55	62.47 ± 6.54	61.42 ± 6.44	0.011
BMI	32.21 ± 5.38	31.24 ± 5.12	32.17 ± 5.40	32.61 ± 5.40	32.82 ± 5.45	0.01
Waist	106.75 ± 13.59	104.26 ± 12.98	106.81 ± 13.64	107.67 ± 13.52	108.27 ± 13.85	0.011
Race	9544 (100%)	2386 (25%)	2386 (25%)	2386 (25%)	2386 (25%)	<0.001
Black	1774 (18.6%)	361 (3.8%)	402 (4.2%)	449 (4.7%)	562 (5.9%)	
Hispanic	678 (7.1%)	130 (1.4%)	147 (1.5%)	180 (1.9%)	221 (2.3%)	
White	5985 (62.7%)	1544 (16.2%)	1574 (16.5%)	1502 (15.7%)	1365 (14.3%)	
Other	1107 (11.6%)	351 (3.7%)	263 (2.8%)	255 (2.7%)	238 (2.5%)	
Education	9544 (100%)	2386 (25%)	2386 (25%)	2386 (25%)	2386 (25%)	<0.001
< HS	1383 (14.5%)	299 (3.1%)	301 (3.2%)	348 (3.6%)	435 (4.6%)	
HS	2529 (26.5%)	626 (6.6%)	613 (6.4%)	646 (6.8%)	644 (6.7%)	
SC	3129 (32.8%)	783 (8.2%)	786 (8.2%)	789 (8.3%)	771 (8.1%)	
More	2503 (26.2%)	678 (7.1%)	686 (7.2%)	603 (6.3%)	536 (5.6%)	
HbA1c	8.29 ± 1.05	7.69 ± 0.65	7.99 ± 0.74	8.43 ± 0.89	9.06 ± 1.25	<0.001
Duration of DM	10.78 ± 7.57	11.18 ± 7.75	11.05 ± 7.65	10.84 ± 7.58	10.07 ± 7.24	0.001
SBP	136.28 ± 17.00	135.49 ± 16.54	136.1 ± 16.57	136.7 ± 17.27	136.8 ± 17.60	0.038
Heart rate	72.58 ± 11.70	71.08 ± 11.38	72.22 ± 11.75	72.91 ± 11.92	74.12 ± 11.52	0.115
cholesterol	183.27 ± 41.82	178.36 ± 38.74	181.5 ± 40.60	183.4 ± 40.45	189.7 ± 46.30	<0.001
TRIG	190.4 ± 149.7	168.8 ± 109.5	182.0 ± 125.5	194.8 ± 145.9	216.1 ± 198.5	<0.001
HDL	41.86 ± 11.56	43.43 ± 11.73	42.08 ± 11.62	41.24 ± 11.32	40.69 ± 11.37	0.127
CVD history	3335 (34.9%)	809 (8.5%)	817 (8.6%)	857 (9.0%)	852 (8.9%)	0.354
CHF history	439 (4.6%)	105 (1.1%)	96 (1.0%)	126 (1.3%)	112 (1.2%)	0.204
Smoke	9544 (100%)	2386 (25%)	2386 (25%)	2386 (25%)	2386 (25%)	<0.001
Current	1323 (13.92%)	289 (3%)	287 (3%)	331 (3.5%)	416 (4.4%)	
Ever	4246 (44.5%)	1084 (11.4%)	1079 (11.3%)	1053 (11%)	1030 (10.8%)	
No smoke	3975 (41.6%)	1013 (10.6%)	1020 (10.7%)	1002 (10.5%)	940 (9.8%)	
Alcohol	0.97 ± 2.70	1.11 ± 2.86	1.07 ± 2.91	0.80 ± 2.33	0.91 ± 2.62	<0.001
Thiazide	2651 (27.8%)	688 (7.2%)	689 (7.2%)	657 (6.9%)	617 (6.5%)	0.065
ARBs	1591 (16.7%)	445 (4.7%)	423 (4.4%)	394 (4.1%)	329 (3.4%)	<0.001
ACE-inhibitors	5217 (54.7%)	1274 (13.3%)	1292 (13.5%)	1333 (14.0%)	1318 (13.8%)	0.318
Beta-blocker	2882 (30.2%)	709 (7.4%)	711 (7.4%)	752 (7.9%)	710 (7.4%)	0.451
Any insulin	3348 (35.1%)	872 (9.1%)	851 (8.9%)	842 (8.8%)	783 (8.2%)	0.045
Statin	6086 (63.8%)	1635 (17.1%)	1544 (16.2%)	1533 (16.1%)	1374 (14.4%)	<0.001
IG	4760 (49.9%)	946 (9.9%)	1173 (12.3%)	1260 (13.2%)	1381 (14.5%)	<0.001

*Values are mean ± SD for continuous variables and n (%) for categorical variables; BMI, body mass index; HS, high school; SC, some college; SBP, systolic blood pressure; DM, diabetes mellitus; TRIG, triglyceride; HDL, high-density lipoprotein; CVD, cardiovascular disease; ARBs, angiotensin receptor blocker; ACE, angiotensin-converting enzyme; IG, intensive glycaemia control; Q, quartile*.

Individuals with higher variability of HbA1c than those with a lower variability of HbA1c tended to be younger, had a higher body mass index (BMI), waist, cholesterol, triglyceride (TRIG), and smoke more frequently, had a shorter duration of DM. Over a median follow-up period of 4.85 years, 943, 594, 268, 414 individuals, respectively, were adjudicated as having the primary outcome, all-cause mortality, CVD mortality, and heart failure, respectively.

**Figure 2:**
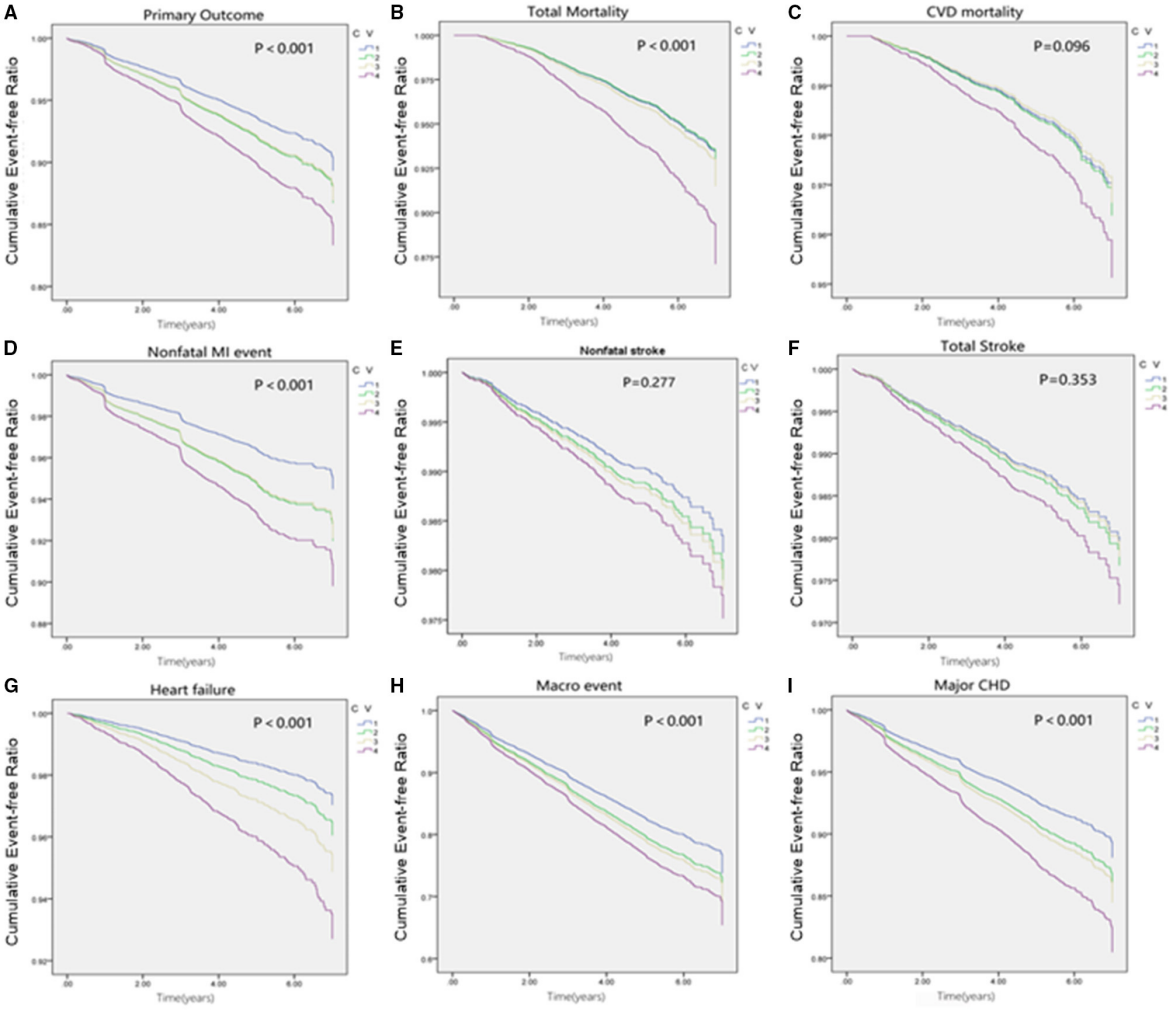
Cumulative survival of outcomes grouped by quartiles of HbA1c fluctuation. **(A)** Cumulative survival of Primary outcome grouped by quartiles of HbA1c fluctuation. **(B)** Cumulative survival of Total mortality grouped by quartiles of HbA1c fluctuation. **(C)** Cumulative survival of CVD mortality grouped by quartiles of HbA1c fluctuation. **(D)** Cumulative survival of Non-fatal MI event grouped by quartiles of HbA1c fluctuation. **(E)** Cumulative survival of Nonfatal stroke grouped by quartiles of HbA1c fluctuation. **(F)** Cumulative survival of Total stroke grouped by quartiles of HbA1c fluctuation. **(G)** Cumulative survival of CHF grouped by quartiles of HbA1c fluctuation. **(H)** Cumulative survival of Macro event grouped by quartiles of HbA1c fluctuation. **(I)** Cumulative survival of Primary outcome grouped by quartiles of HbA1c fluctuation.

The cumulative incidence curves for the cardiovascular outcome and all-cause mortality within subgroups defined by the variability of HbA1c are presented in Figure 2. In our fully adjusted Cox model, visit-to-visit variability of HbA1c including CV, SD, and VIM was associated with primary outcome, all-cause mortality, CVD death, nonfatal MI event, total stroke, heart failure, microvascular events, and major coronary disease but not with non-fatal stroke (Table 2). HR of primary and secondary outcomes are summarized in Table 3 when HbA1c was calculated as a category. We found that the risk of CVD outcome increased with higher levels of HbA1c variability.

**Table 2:** Visit-to-visit variability of HbA1Cas a continuous variable and cardiovascular outcomes.

**CVD outcome**	**Model 1**		**Model 2**		**Model 3**		**Model 4**	
	**HR (95%CI)**	**P**	**HR (95%CI)**	**P**	**HR (95%CI)**	**P**	**HR (95%CI)**	**P**
**Primary outcome**								
SD	1.31 (1.23–1.38)	<0.001	1.29 (1.22–1.36)	<0.001	1.25 (1.17–1.32)	<0.001	1.22 (1.15–1.31)	<0.001
CV	1.27 (1.20–1.35)	0.004	1.26 (1.18–1.33)	<0.001	1.23 (1.54–1.31)	0.071	1.20 (1.11–1.29)	<0.001
VIM	2.12 (1.81–2.49)	<0.001	2.06 (1.75–2.42)	<0.001	1.87 (1.59–2.20)	<0.001	1.79 (1.48–2.16)	<0.001
**Total mortality**								
SD	1.35 (1.26–1.45)	<0.001	1.32 (1.23–1.41)	<0.001	1.29 (1.20–1.39)	<0.001	1.31 (1.21–1.42)	<0.001
CV	1.32 (1.23–1.42)	<0.001	1.28 (1.19–1.38)	<0.001	1.27 (1.18–1.37)	<0.001	1.28 (1.17–1.40)	<0.001
VIM	2.34 (1.92–2.85)	<0.001	2.18 (1.78–2.66)	<0.001	2.05 (1.67–2.52)	<0.001	2.15 (1.70–2.71)	<0.001
**CVD mortality**								
SD	1.34 (1.20–1.49)	<0.001	1.30 (1.17–1.45)	<0.001	1.28 (1.15–1.43)	<0.001	1.26 (1.11–1.43)	0.001
CV	1.27 (1.14–1.43)	<0.001	1.24 (1.10–1.39)	<0.001	1.24 (1.10–1.40)	0.001	1.19 (1.03–1.37)	0.017
VIM	2.28 (1.69–3.07)	<0.001	2.14 (1.57–2.90)	<0.001	2.01 (1.47–2.75)	<0.001	1.92 (1.33–2.77)	<0.001
**Non-fatal MI event**								
SD	1.30 (1.21–1.39)	<0.001	1.29 (1.20–1.38)	<0.001	1.24 (1.16–1.33)	<0.001	1.25 (1.16–1.36)	<0.001
CV	1.27 (1.18–1.37)	<0.001	1.26 (1.17–1.36)	<0.001	1.23 (1.14–1.33)	<0.001	1.24 (1.13–1.35)	<0.001
VIM	2.09 (1.72–2.55)	<0.001	2.05 (1.68–2.51)	<0.001	1.85 (1.51–2.27)	<0.001	1.90 (1.51–2.39)	<0.001
**Non-fatal stroke**								
SD	1.40 (1.24–1.58)	<0.001	1.39 (1.23–1.58)	<0.001	1.33 (1.18–1.51)	<0.001	1.17 (0.99–1.38)	0.063
CV	1.40 (1.23–1.60)	<0.001	1.40 (1.22–1.60)	<0.001	1.36 (1.18–1.56)	<0.001	1.16 (0.97–1.39)	0.095
VIM	2.60 (1.84–3.66)	<0.001	2.55 (1.80–3.61)	<0.001	2.25 (1.57–3.21)	<0.001	1.55 (0.98–2.47)	0.064
**Total stroke**								
SD	1.42 (1.27–1.59)	<0.001	1.42 (1.26–1.59)	<0.001	1.37 (1.21–1.54)	<0.001	1.22 (1.04–1.42)	0.012
CV	1.43 (1.26–1.62)	<0.001	1.43 (1.26–1.62)	<0.001	1.39 (1.22–1.59)	<0.001	1.22 (1.03–1.43)	0.019
VIM	2.71 (1.96–3.74)	<0.001	2.69 (1.94–3.73)	<0.001	2.41 (1.72–3.37)	<0.001	1.74 (1.13–2.68)	0.011
**CHF**								
SD	1.47 (1.36–1.58)	<0.001	1.42 (1.31–1.54)	<0.001	1.39 (1.28–1.51)	<0.001	1.40 (1.27–1.53)	<0.001
CV	1.43 (1.31–1.55)	<0.001	1.37 (1.26–1.49)	<0.001	1.36 (1.25–1.49)	<0.001	1.35 (1.22–1.50)	<0.001
VIM	2.95 (2.38–3.67)	<0.001	2.72 (2.17–3.41)	<0.001	2.54 (2.01–3.21)	<0.001	2.59 (1.99–3.37)	<0.001
**Macro event**								
SD	1.20 (1.15–1.24)	<0.001	1.18 (1.13–1.26)	<0.001	1.15 (1.10–1.19)	<0.001	1.15 (1.09–1.20)	<0.001
CV	1.17 (1.24–1.22)	<0.001	1.16 (1.11–1.20)	<0.001	1.13 (1.09–1.18)	<0.001	1.13 (1.08–1.19)	<0.001
VIM	1.64 (1.47–1.84)	<0.001	1.59 (1.42–1.78)	<0.001	1.47 (1.30–1.65)	<0.001	1.48 (1.29–1.69)	<0.001
**Major CHD**								
SD	1.23 (1.17–1.30)	<0.001	1.22 (1.15–1.28)	<0.001	1.18 (1.12–1.25)	<0.001	1.20 (1.12–1.27)	<0.001
CV	1.21 (1.14–1.28)	0.034	1.19 (1.13–1.26)	<0.001	1.18 (1.11–1.28)	<0.001	1.18 (1.11–1.27)	<0.001
VIM	1.80 (1.54–2.10)	<0.001	1.75 (1.50–2.04)	<0.001	1.61 (1.38–1.88)	<0.001	1.67 (1.40–1.99)	<0.001

*Model 1, adjusted for sex, age, race, duration of diabetes mellitus at baseline. Model 2, further adjusted for level of education, smoking, alcohol abuse, BMI, waist at baseline. Model 3 included systolic blood pressure, heart rate, cholesterol, CVD history, insulin usage, antihypertension or lipid-lowering medication. Model 4, accounted for baseline HbA1c*.

**Table 3:** Visit-to-visit variability of HbA1C as a categorical variable and cardiovascular outcomes.

	**Model 1**		**Model 2**		**Model 3**		**Model 4**	
	**HR (95%CI)**	***P***	**HR (95%CI)**	***P***	**HR (95%CI)**	***P***	**HR (95%CI)**	***P***
**Primary outcome**								
Q1	reference		reference		reference		reference	
Q2	1.30 (1.07–1.59)	0.008	1.30 (1.07–1.58)	0.009	1.29 (1.06–1.57)	0.012	1.26 (1.03–1.54)	0.022
Q3	1.41 (1.16–1.71)	0.001	1.37 (1.13–1.67)	0.002	1.30 (1.07–1.58)	0.009	1.24 (1.01–1.52)	0.041
Q4	1.95 (1.61–2.35)	<0.001	1.88 (1.55–2.28)	<0.001	1.76 (1.45–2.14)	<0.001	1.61 (1.29–2.00)	<0.001
**Total mortality**								
Q1	reference		reference		reference		reference	
Q2	0.99 (0.78–1.28)	0.977	0.99 (0.78–1.28)	0.977	0.99 (0.77–1.27)	0.941	0.99 (0.77–1.27)	0.933
Q3	1.18 (0.92–1.50)	0.187	1.18 (0.92–1.50)	0.187	1.08 (0.84–1.38)	0.563	1.07 (0.83–1.38)	0.592
Q4	1.87 (1.48–2.35)	<0.001	1.87 (1.48–2.35)	<0.001	1.67 (1.32–2.12)	<0.001	1.66 (1.27–2.17)	<0.001
**CVD mortality**								
Q1	reference		reference		reference		reference	
Q2	1.06 (0.74–1.52)	0.763	1.04 (0.72–1.50)	0.844	1.07 (0.74–1.55)	0.711	1.04 (0.72–1.51)	0.828
Q3	1.51 (0.80–1.66)	0.449	1.07 (0.74–1.55)	0.707	1.03 (0.71–1.48)	0.885	0.96 (0.66–1.40)	0.830
Q4	1.75 (1.23–2.47)	0.002	1.60 (1.13–2.27)	0.008	1.61 (1.13–2.29)	0.008	1.41 (0.94–2.12)	0.096
**Non-fatal MI event**								
Q1	reference		reference		reference		reference	
Q2	1.47 (1.15–1.88)	0.002	1.49 (1.16–1.89)	0.002	1.47 (1.15–1.88)	0.002	1.47 (1.15–1.88)	0.002
Q3	1.54 (1.20–1.97)	0.001	1.52 (1.19–1.96)	0.001	1.45 (1.13–1.87)	0.004	1.45 (1.12–1.88)	0.005
Q4	2.05 (1.60–2.61)	<0.001	2.01 (1.57–2.57)	<0.001	1.89 (1.48–2.43)	<0.001	1.89 (1.43–2.48)	<0.001
**Non-fatal stroke**								
Q1	reference		reference		reference		reference	
Q2	1.33 (0.79–2.23)	0,285	1,34 (0.80–2.26)	0.265	1.29 (0.77–2.16)	0.342	1.15 (0.68–1.95)	0.594
Q3	1.69 (1.03–2.79)	0.039	1.70 (1.03–2.81)	0.037	1.56 (0.94–2.58)	0.082	1.21 (.072–2.04)	0.478
Q4	2.53 (1.57–4.08)	<0.001	2.52 (1.55–4.08)	<0.001	2.25 (1.38–3.67)	0.001	1.38 (0.78–2.41)	0.277
**Total stroke**								
Q1	reference		reference		reference		reference	
Q2	1.24 (0.77–1.98)	0.383	1.25 (0.78–2.01)	0.351	1.20 (0.74–1.93)	0.458	1.08 (0.67–1.74)	0.765
Q3	1.42 (0.89–2.26)	0.140	1.43 (0.90–2.29)	0.129	1.31 (0.82–2.10)	0.254	1.02 (0.63–1.67)	0.928
Q4	2.32 (1.50–3.60)	<0.001	2.33 (1.50–3.62)	<0.001	2.09 (1.33–3.28)	0.001	1.28 (0.76–2.17)	0.353
**CHF**								
Q1	reference		reference		reference		reference	
Q2	1.37 (0.99–1.90)	0.061	1.37 (0.99–1.90)	0.061	1.35 (0.97–1.88)	0.072	1.35 (0.97–1.88)	0.077
Q3	2.07 (1.52–2.82)	<0.001	2.07 (1.52–2.82)	<0.001	1.77 (1.29–2.42)	<0.001	1.75 (1.27–2.41)	0.001
Q4	3.01 (2.22–4.08)	<0.001	3.01 (2.22–4.08)	<0.001	2.57 (1.88–3.49)	<0.001	2.52 (1.79–3.54)	<0.001
**Macro event**								
Q1	reference		reference		reference		reference	
Q2	1.21 (1.07–1.37)	0.003	1.20 (1.06–1.36)	0.004	1.19 (1.05–1.35)	0.006	1.19 (1.05–1.35)	0.007
Q3	1.34 (1.18–1.52)	<0.001	1.31 (1.16–1.48)	<0.001	1.24 (1.10–1.41)	0.001	1.23 (1.08–1.40)	0.002
Q4	1.55 (1.37–1.75)	<0.001	1.49 (1.32–1.69)	<0.001	1.42 (1.25–1.61)	<0.001	1.39 (1.21–1.60)	<0.001
**Major CHD**								
Q1	reference		reference		reference		reference	
Q2	1.25 (1.05–1.50)	0.014	1.24 (1.04–1.49)	0.018	1.25 (1.04–1.50)	0.016	1.26 (1.05–1.51)	0.013
Q3	1.40 (1.17–1.67)	<0.001	1.36 (1.14–1.63)	0.001	1.31 (1.09–1.57)	0.003	1.33 (1.10–1.60)	0.003
Q4	1.78 (1.49–2.12)	<0.001	1.71 (1.43–2.04)	<0.001	1.66 (1.39–1.99)	<0.001	1.71 (1.40–2.08)	<0.001

*Participants were divided into four quartiles of CV of HbA1c. Model 1, adjusted for sex, age, race, duration of diabetes mellitus at baseline. Model 2, further adjusted for level of education, smoking, alcohol abuse, BMI, waist at baseline. Model 3 included systolic blood pressure, heart rate, cholesterol, CVD history, insulin usage, antihypertension or lipid-lowering medication. Model 4, accounted for baseline HbA1c*.

**Figure 3:**
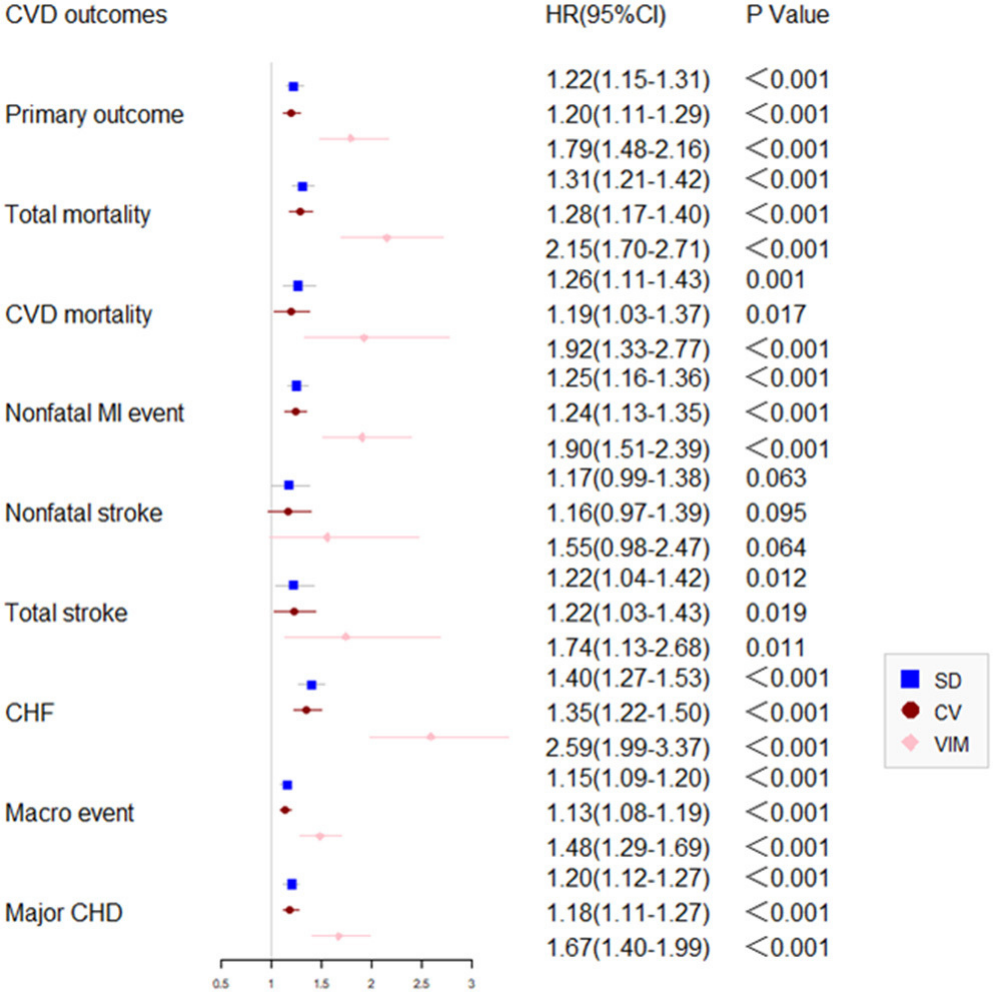
Comparison of primary and secondary outcomes by level of HbA1c variability in the ACCORD cohort.

Compared with participants with the lowest quartile, after adjustment of potential confounding factors, the HR (95% CI) for primary outcome in the second, third, and the highest quartiles of variability of HbA1c were 1.26 (1.03–1.54), 1.24 (1.01–1.52), 1.61 (1.29–2.00), respectively. Similar trends were also noted in all secondary outcomes including non-fatal MI, any and all-cause mortality, major coronary events, and fatal and non-fatal heart failure except for non-fatal stroke (Figure 3). Noticeably, the adjusted HR comparing patients in the highest vs. the lowest quartile CV of HbA1c variability was 1.67 (95% CI 1.28–2.18) for all-cause mortality.

Participants in the highest quartile experienced the highest risk than other groups during 7 years of follow-up (*P* < 0.05).

## Discussion

Previous studies had established HbA1c as an effective index of long-term glycemic control, lower HbA1c persisting at <7% was associated with less risks for diabetes related complications and per 1% higher HbA1c is related to 15–20% higher cardiovascular risk (25, 26). However, whether controlling HbA1c to a normal range by intensive glycemic therapy in patients with T2DM can reduce CVD remains controversial. ACCORD, VADT, and other large studies showed no beneficial effects of intensive glucose therapy targeting for low-level HbA1c (27, 28). Hence, whether the mean HbA1c level is the most appropriate factor to predict the risk for diabetes complication is still questionable. Many researchers highlighted the effects of HbA1c variability on the cardiovascular outcomes.

To our best knowledge, our study was the first one to explore the association between the long-term visit-to-visit fluctuation of HbA1c levels CVD outcomes using ACCORD data. We discovered patients with T2DM with higher variability of HbA1c tended to develop cardiovascular diseases and had a worse prognosis. This observation was consistent after adjusting for the baseline HbA1c and other confounding factors such as demographic characteristics, hypertension, dyslipidemia, smoking, and medications. In accordance with our results, a Chinese study had evaluated the association between HbA1c variability and vascular complication and mortality (11). Moreover, Prentice et al. conducted a retrospective study with T2DM and concluded that visit-to-visit HbA1c fluctuation might predict adverse outcomes (29). A recent review pooled 20 studies with 87,641 participants to investigate on HbA1c fluctuation and vascular complication in type 1 diabetes in T2DM. Particularly, in T2DM, higher HbA1c variability group had higher risk for cardiovascular disease, renal disease, mortality (14). In contrast, the RIACE study shown no association between HbA1c and macrovascular disease. The potential explanation for such disparity may be due to the study design. We collected the multiple HbA1c variability measurements of 7 years, with a median follow-up time of 4.85 years, while the RIACE obtained serial HbA1c values during 2-year period recruitment. Furthermore, our study held distinct baseline HbA1c and degree of HbA1c fluctuation and diabetes duration (17).

Until now, there has not been a standardized method to access HbA1c variability. We selected three measurements including SD, CV, VIM, all of which were independently correlated with the cardiovascular outcomes. Our results showed that the CV is possibly a more robust measure of visit-to-visit in HbA1c rather than average HbA1c levels. Unfortunately, researchers fail to carry out intervention study and conclude the cause-effect of HbA1c variability. The mechanisms linking the higher HbA1c fluctuation to the higher risk of CVD remain unclear and require further biological investigation. One of the hypotheses is that glycemic variability might cause endothelial dysfunction and atherosclerosis induced by inflammatory cytokines and oxidative stress (30–32). Another hypothesis is the “metabolic memory” in vascular cells, by which the cellular transduction system and extra oxygen and nitrogen lead to endothelial damage (33). Moreover, the glucose fluctuation may cause hypoglycemia which poses a threat to cardiovascular systems (34).

There are several limitations to our present study. First, the number and frequency of HbA1c value varied from different participants. To minimize the influences, we use CV and VIM which are independent of average of HbA1c levels. Second, the most of our participants had a DM duration of over 10 years, thus, it is uncertain whether our conclusion could be generalized to those with a shorter duration of DM. Third, we failed to conduct the trend of HbA1c variability. Whether decreasing the HbA1c variability could reduce cardiovascular outcomes and all-cause mortality remains obscure and needs further investigation.

## Conclusions

In conclusion, data from a 7-year follow-up cohort has revealed that high variability of HbA1c is an independent risk for cardiovascular outcomes of the ACCORD study. Moreover, it seems reasonable to include HbA1c variability as potential target in the routine management of T2DM.

## Data Availability Statement

The data of the Action to Control Cardiovascular Risk in Diabetes study is available free of charge from the NHLBI Biologic Specimen and Data Repository Information Coordinating Center, the datasets used during the current study are available from the corresponding authors on reasonable request.

## Ethics Statement

The studies involving human participants were reviewed and approved by the Fifth Affiliated Hospital, Sun Yat-sen University. The patients/participants provided their written informed consent to participate in this study.

## Author Contributions

YH and DH planned and interpreted the data. YH, DH, and Y-QH drafted the manuscript. Q-YZ and YC performed the statistical analyses. YH is the guarantor of the present study and took responsibility for the integrity and the accuracy of the data analysis. All authors read and revised the content and approved the final manuscript.

## Funding

This work was supported by the Zhuhai Science and Technology Planning Project (ZH22036201210055PWC).

## Conflict of Interest

The authors declare that the research was conducted in the absence of any commercial or financial relationships that could be construed as a potential conflict of interest.

## Publisher's Note

All claims expressed in this article are solely those of the authors and do not necessarily represent those of their affiliated organizations, or those of the publisher, the editors and the reviewers. Any product that may be evaluated in this article, or claim that may be made by its manufacturer, is not guaranteed or endorsed by the publisher.
